# No home for poor men: a comparative study of household debt and homeownership in Denmark and Turkey

**DOI:** 10.1007/s10901-022-09930-8

**Published:** 2022-04-09

**Authors:** Suheyla Turk, Burag Gurden

**Affiliations:** 1 Department of Human Geography, Geocentrum I, Sölvegatan 10, Lund, Sweden; 2 Department of Geography, Durham University, DH1 3LE Durham, UK

**Keywords:** asset-based welfare, homeownership, wealth inequalities, Denmark, Turkey

## Abstract

Homeownership rates have declined in several countries including Denmark and Turkey since 2010. A majority of the decline in homeownership has been observed among low income holders. This variation finding comparative case study compares similar patterns of neoliberal housing policies to examine wealth inequalities based on homeownership despite fundamental differences in housing markets and welfare state provision. The comparison of Denmark and Turkey reveals similar adoption of policies that support financialization as a strategy to recover from financial crises. This paper examines how states have supported financialization with policies that allowed deregulations in the housing market to create an enabling environment for construction and real estate-specific growth, and how neoliberal housing policies positioned homeownership, a wealth symbol, as the core tenet of asset-based welfare that increased wealth inequalities. The outcome of this paper shows that neoliberal housing policies have generated new forms of inequality between low and high-income earners to access housing in both countries in different ways to produce a similar outcome.

## Introduction

Homeownership rates have been falling in many countries such as in the UK, US, Australia, Denmark and Turkey since 2010 and household debts have been increasing that cannot alone be ascribed to the shock effect of the 2007 financial crisis (Goodman & Mayer, 2018; McKee, 2012). Strategies to overcome the financial crises have included neoliberal housing policies that have increased home purchases (Andersen & Winther, 2010). However, strategies have also led to the development of asset-based welfare, although homeownership of low-income holders has declined (Doling & Ronald, 2010; Ronald, 2015; Stebbing & Spies-Butcher, 2016).

People have become attracted to becoming homeowners with the use of home loan promotions through increased mortgage volumes of banks, a characteristic of actually existing neoliberalism (Brenner & Theodore, 2017). These forms of housing financialization have been coupled with tax incentives, which have led to increased wealth inequalities among people (Wind et al., 2019). This study incorporates the term wealth inequality, as put forward by Piketty (2015), that is different from income inequalities that is measured with Gini coefficients^1^ (Balestra & Tonkin, 2018). Wealth inequality is explained as the return capital of top 1% to 10% income holders, from financial and non-financial assets, which grow larger than the rest of the society (Balestra & Tonkin, 2018; Piketty, 2015; Ryan-Collins et al., 2017). For example, the share of top 10% income holders in OECD countries has 52% of net wealth of countries (Balestra & Tonkin, 2018; Ryan-Collins et al., 2017). However, the top 10% own only 24% of the total income of people living in OECD countries in 2015; a (Balestra & Tonkin, 2018; Ryan-Collins et al., 2017). One of the reasons for this difference is the wealth gained by homeownership, being one of the main contributers of wealth inequality in some countries (Balestra & Tonkin, 2018; Ryan-Collins et al., 2017). Financialization is regarded in this study as a process led by financial institutions of transforming homeownership into an asset that is facilitated through neoliberal housing policies consisting of the expansion of long-term payments, mortgage programs and establishment of real estate institutions to subsidize housing for profit making (Byrne & Norris, 2019). Neoliberal housing policies are explained in this study as tax incentives and national support to home ownership with mortgage loans resulting in constant appreciation of housing values that benefit housing builders and homeowners to increase profits.

Neoliberal housing policies facilitate financialization of housing, which is a centre point of wealth inequalities based on return capital from non-financial assets with income from homeownership transactions and rents (Fuller et al., 2020; Petach, 2018; Piketty, 2015; Ryan-Collins et al., 2017). Non-financial assets gathered from the increases of housing and rent values have become income to homeowners on the one hand (Fuller et al., 2020; Ryan-Collins et al., 2017). On the other hand, increasing housing expenses have led to affordability problems, and indebtedness for low-income groups who borrow mortgages to purchase homes. Therefore, the metaphor no home for poor men refers to the result of housing financialization that hinders low-income holders to access housing with lower rent and purchase prices, while providing generous opportunities to higher income holders who have easy access to mortgage loans (Crawford & McKee, 2018; Fuller et al., 2020; Piketty, 2015; Wood, 2019).

This paper tries to answer how and why neoliberal housing policies have changed homeownership rates among different income holders in the surge of household debts in Denmark and Turkey. Denmark and Turkey are similar and different in their relationship between distribution of wealth^2^ based on homeownership and debt among different income groups. Homeownership rates in Denmark and Turkey are lower in comparison to other countries situated in the European continent. Denmark and Turkey have contrasting but marginal features among other OECD countries in terms of the share of household debts and income inequalities. Across other OECD countries in 2015, Denmark had a highest mortgage debt rate and one of the lowest rates of income inequality; while Turkey had the second lowest mortgage debt rates and one of the highest rates of income inequality (OECD, 2020c; Whitehead & Williams, 2017).

In both Denmark and Turkey, housing financialization has emerged leading to economic growth and increasing housing prices, although these states support financialization differently. In Denmark, financialization has been supported indirectly with eased mortgage borrowing causing a growth in household debts and housing prices. Denmark has the second lowest share of housing outright among OECD countries due to the fact that many of the mortgage borrowers sell homes before paying back their loans to gain profit and has one of the highest rates of mortgage debt.

Turkey has one of the lowest shares of mortgage usage and debt among OECD countries because family equity is often helpful to people for purchasing a home. However, new financial institutions were established to ease and increase homeownership. The Turkish state began to directly support housing financialization by the establishment of Emlak Bank, in 1946 (Gurbuz, 2001). Emlak Bank was a public bank that specialized in real estate and gave mortgages to housing cooperatives and individuals (Gurbuz, 2001). Also, homeownership was linked to the welfare system with provision of housing to be purchased through a state-owned affordable housing (AH) production institution, Toplu Konut Idaresi (TOKI) (Bayirbağ, 2013; TOKI, 2012). In 2004, TOKI was authorized to make profits through neoliberal housing policies including revenue sharing agreements with private companies for housing production for middle- and high-income groups (TOKI, 2012).

The next section presents the arguments shaping a critical discussion of financialization, pertaining to housing systems of the case countries, Denmark and Turkey, based on their welfare structure, tenure distribution and housing markets. The third section explains the method and data, and the fourth section presents findings regarding the usage of mortgage loans, promotion of homeownership and wealth inequality. The last part consists of discussion and conclusion.

### Arguments of the paper

Neoliberal housing policies aim for and rely upon economic growth based on financialization of housing leading to income inequalities. As pointed out by Sonia Alves and Andersen (2019) policies are developed with direct and indirect state support through deregulations in housing markets. Financial crisis years are selected for analysis of this paper, due to the decline in economic growth and increase in unemployment rates that led states to develop fast recovery policies (Claessens et al., 2014). Neoliberal housing policies are analysed considering financial crisis years that have impacts on Denmark in 2007. In Turkey, the financial crisis of 2001 and the currency crisis of 2018 have deeply affected society. Arguments made in this paper build upon the following recent neoliberal housing policy developments supporting financialization.

First, states have supported financialization with policies that allowed deregulations in the housing market to create an enabling environment for construction and real estate-specific growth. The argument was supported with OECD statistical data showing that two countries more than doubled the number of housing construction between 2011 and 2018 (OECD, 2020a).While Turkey had the fourth highest number of produced housing units among OECD countries in 2018, Denmark more than tripled the number of housing units produced in 2011 from 8,000 to 30,000 in 2020 (OECD, 2020a). Hence, real estate specific growth proved effective in lifting economic growth, and coupled with financialization led to growing house prices, household debts and inequalities (Di Feliciantonio, 2016). Stiglitz (2012, p.77) elaborates on the government’s role in challenging inequality as:

“*Government today plays a double role in our current inequality: it is partly responsible for the inequality in before-tax distribution of income, and it has taken a diminished role in “correcting” this inequality through progressive tax and expenditure policies*”.

Second, neoliberal housing policies have positioned homeownership, as a perceived wealth symbol, as the core tenet of asset-based welfare causing wealth inequalities (Crawford & McKee, 2018; Fuller et al., 2020; Piketty, 2015). Doling and Ronald (2010) explain the desire of becoming a homeowner as being challenged by concern of mortgage debt responsibility. The concluding statements of their study comment on the weakened social welfare system resulting from equity-based welfare practises, that align with the hidden factor of wealth inequality, which are in parallel with the findings of this paper. Increasing prices of housing and reductions in taxation led to increasing housing prices that assist homeowners to create wealth but cause affordability problems for low-income holders (Ryan–Collins, 2018; Wood, 2019).

## Background: Connection of neoliberal housing policies in welfare

Welfare systems are state-supported social services that help individual well-being, which are financed by sources collected but independent of the private market and related competitiveness (MacLeavy, 2016; Malpass, 2008). State control impacts distribution of tenure by providing financial incentives such as subsidizing tenants living in rental housing; regulating rent rates to keep it below the market levels to balance the distribution of wealth (Forrest, 2018; Fuller et al., 2020; Malpass, 2008). However, asset-based welfare practices have been promising future financial security to middle- and high-income holders with income gains in return from housing assets regardless of welfare systems, as explained by Doling and Ronald (2010, p.165);

*“the principle underlying an asset-based approach to welfare is that, rather than relying on state-managed social transfers to counter the risks of poverty, individuals accept greater responsibility for their own welfare needs by investing in financial products and property assets which increase in value over time’’*.

Homeownership rates among low-income holders have been declining in the two countries. In Denmark, homeownership rates declined from 67.4% in 2006 to 60.5% in 2018 (Statbank, 2019a). In the following year, the national bank of Denmark has had a − 0.5% interest rate and private banks in Denmark offer 20-year mortgage loans with no interest (DanmarksNationalbank, 2021a). In Turkey, homeownership rate declined from 60.9% in 2006 to 59% in 2018 (TUIK, 2021a). Meanwhile, the Turkish government to reduced interest rates of mortgage loans via Turkish Central Bank in the following year, 2019, to promote homeownership.

### Welfare systems and income taxation in Denmark and Turkey

Complementary income taxes and social spending in welfare are intended to restrain income inequality gaps through the redistribution of wealth by tax returns (Stiglitz, 2012). Denmark has a social-democratic welfare system providing equal social services. Danish municipalities use the funds received from income taxes to provide welfare services such as schools, hospitals, jobs and unemployment benefits for all citizens (Esping-Andersen, 2016). Income taxes are used to redistribute capital to subsidize social services to the employed and unemployed Danish public (Andersen, 2011; Esping-Andersen, 2016; OECD, 2018a).

Particularly since the financial crisis of 2007, more austerity measures have been used to recover national economies by disciplining urban poor with workfare policies while contingently providing public subsidies (Theodore, 2020). In 2007, municipalities were transferred to the assistance of employment services in 2007 from regions and the power of unions diminished in 2009 (Christiansen & Klitgaard, 2009; Harsløf & Ulmestig, 2013). Municipalities have been providing more funding to subsidize unemployed people who are actively seeking employment or attending vocational education. Unemployment subsidies provided for inactive unemployed that were reduced from 50 to 35% in 2009 (Harsløf & Ulmestig, 2013). One of the negative results of change in the amount of funding is a limited budget for the local municipalities that have had a high number of unemployed people for a long period of time. Therefore, these municipalities have less funding to be allocated for social projects, such as supporting low-income housing projects or creating solutions to solve unemployment problems (Christiansen & Klitgaard, 2009; Harsløf & Ulmestig, 2013).

**Fig. 1:**
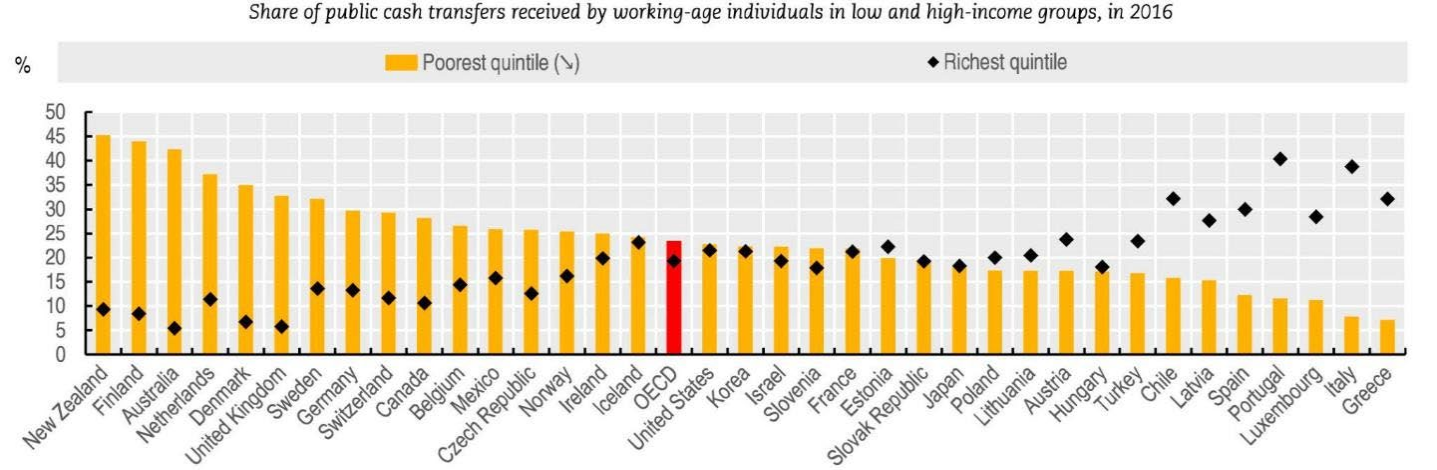
The share of social benefits going to low- and higher-income households in OECD in 2019, p. 105 (OECD, 2019)

Turkey has a rudimentary welfare system that does not have full employment traditions and has informal-security because of high shares of rural and informal economic activities (l’emploi & Iguarán, 2011; Powell & Yörük, 2017). The welfare system of Turkey is based on fragile institutional relationships impacted by political and economic interventions; and a low share of social spending to be used for funding unemployed low-income groups because the system primarily provides welfare benefits for employed populations (Bugra & Candas, 2011; l’emploi & Iguarán, 2011; Powell & Yörük, 2017). As shown in Fig. 1, low-income households receive 16% of cash subsidy shares, while highest income households were provided 25% of public cash transfers in 2016 (OECD, 2019).

Regardless, social benefits given to the unemployed have increased since 2004 in Turkey. In 2004, people with a household income less than 30% of formal minimum income were considered eligible to access free health service without having social security (LawNo:25678, 2004). The Social Assistance Fund has been used to help people without health care and pension benefits since 2004; they are given green cards to receive benefits from the health service (LawNo:25678, 2004). In 2015, 15.49% of household members in Turkey without social security benefits from employment, approximately 3 million people, received subsidies from the Social Assistance Fund (ACSHB, 2017). In 2017, subsidies more than doubled in comparison to subsidies given in 2015 (ASPB, 2020).

**Fig. 2:**
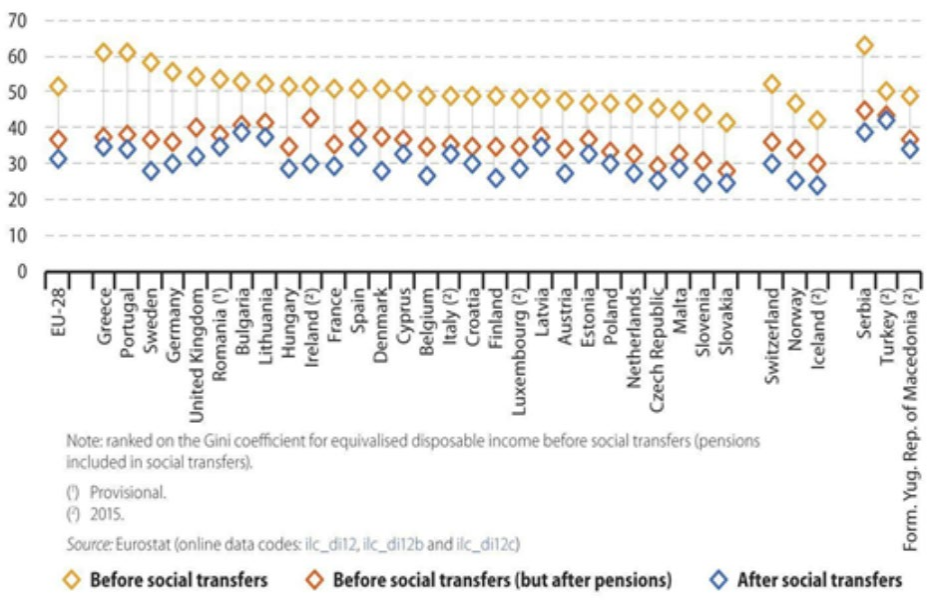
Income inequality before and after social transfers in countries in 2016 by Gini Coefficient levels, p. 24 (Eurostat, 2018)

However, income inequality in Turkey has not been affected positively from neither social security benefits nor tax returns. Esping-Andersen (2016) argues that income inequality should be considered before and after taxes to understand the basic success of social distribution in welfare systems. Figure 2 is supportive of the argument of Esping-Andersen (2016) illustrating the difference in social transfers in income of Danish and Turkish citizens before taxes, with almost the same Gini coefficient levels in 2016. After social transfers by taxes, Turkey had a Gini coefficient of 41, while it was 26 in Denmark showing that social transfers through taxation in Turkey have not distributed enough to cope with income inequalities (Eurostat, 2018).

The remarkable difference in income inequalities between these two countries is also related to the distribution of tax returns. In Turkey and Denmark, pensioners receive the highest portion of social benefits^3^ (Eurostat, 2018). Unemployed and low-income holders receive the second highest share of welfare spending in Denmark, while Turkey provides the lowest share (Eurostat, 2018). Commonly, income taxes have been reduced since the mid-1990 s in both countries. Denmark had the second highest tax to GDP ratio (46%) in 2017 of all OECD countries (OECD, 2018a). While in Turkey, the ratio of tax-to-GDP was 24.4% in 2018 and the OECD average was 34.3% (OECD, 2018a).

Furthermore, taxation rates of income reduced, between 1993 and 2019, in these two countries. In Denmark, the highest income holder paid 68.7% of their salary as income tax in 1993 which fell to 56.1% in 2010 (Skatteministeriet, 2019). Since 2018 the highest income holder has been paying 56.5% (Skatteministeriet, 2019). Average income tax was 50% in the mid-80 s, reduced to 33% in the mid-90 s and then to 30% in 2003 (OECD, 2018a). Tax promotion based on mortgage interest rates is an indicator of an indirect support for financialization. Income taxation in Denmark has had an interest rate deduction based on the mortgage debts of citizens since 1987 (Skatteministeriet, 2019; tax.dk, 2019). Since 2009, if a high-income single-person borrows up to 50,000 DKK, the individual will pay 26.6% in income tax, instead of 42.7% (Skatteministeriet, 2019; tax.dk, 2019).

In Turkey, income taxation has changed many times but has not included tax reductions of mortgage loans. Therefore, there is no interest rate deduction from income taxes for being a homeowner with a mortgage. Marginal income taxation increased from 33.7% in 1988 to 42.4% in 1992; then income tax was reduced to 34% in 1994 (Dorlach, 2015). Between 2004 and 2005, income tax declined to 19.3%, the lowest rate it has been since the 1980s (Dorlach, 2015). In 2017 the highest income holder paid 35% of income tax and the average income tax was 23.8% (LawNo:193/103, 2017).

### Tenure distribution and housing markets in Denmark and Turkey

Housing policies, including subsidies and rent control in housing markets, impact the rate of tenancy types (Crawford & McKee, 2018). Homeownership is less promoted by state integrated housing markets having large stock of non-profit housing in comparison to countries that have integrated housing markets (Sónia Alves, 2017). Denmark has a social democratic welfare system and an integrated housing market, with state-controlled rent levels to maintain lower rent amounts than the market rates (Scanlon et al., 2015). The Danish housing market consists of private rental housing for profit, publicly subsidised housing not for profit, owner occupied and cooperative housing (Sónia Alves, 2017). In 1993, rent controls were deregulated; only for homes produced before 1991 were controlled to keep the rent rates lower than market levels (Andersen & Winther, 2010).

The Danish mortgage system was established in 1797 (Wood, 2019). Private mortgage banks provide loans not only to individuals but also to the non-profit housing sector producing affordable rental housing (Wood, 2019). Individual mortgage usage has been promoted with a growth of borrowing mortgage to housing value amount ratio from 40% to 80% in 1982 and with an extension for payback period of mortgages in 1992 (Wood, 2019). In 1992, the Danish government allowed mortgage banks to increase the mortgage repayment period from 20 to 30 years. Furthermore, an introduction of an amortisation free interest-only loan home purchase promotion (afdragsfrie lån, in Danish) has led to a rapid price increase of homes since 2003 (Andersen & Winther, 2010). These interest-only loans comprise 50% of all borrowed loans regardless of their high-risk structure (Bäckman & Khorunzhina, 2019).

Denmark and Turkey have low levels of homeownership and policies target to increase the rate of homeownership in both countries. Denmark had the fourth lowest homeownership rate of 60.5% in 2020 in comparison to EU countries (Eurostat, 2021a). Publicly subsidised rental housing consists of 20% of housing stock in 2018 ((Eurostat, 2021a). In contrast, in Turkey, almost all policies support homeownership instead of providing publicly subsidised affordable rental housing (Turk & Altes, 2010). However, similar to Denmark, Turkey has low rates of homeownership.

Turkey has a dual housing market including formal and informal housing units (Kortes and Turk, 2010). Turkey has the fifth lowest home ownership rate of 59% in 2020, after Switzerland, Germany, and Austria (Eurostat, 2021a; Özdemir, 2011). Private rental housing had a large 28% share of the national housing stock in 2018 (Eurostat, 2021a; Malpass, 2008; Özdemir, 2011). Informal housing units in addition to formal housing shape dual housing systems of countries that have rudimentary welfare systems (Ajzenstadt & Gal, 2010; l’emploi & Iguarán, 2011; Powell & Yörük, 2017). Housing is claimed as an asset in the form of rental income or capital gain by transfer of homeownership in dual housing markets (Bengtsson, 2007; Doling & Ronald, 2010). In Turkey, the state regulates the maximum annual rise in rents but does not require rent levels to be lower than the market rate for low-income groups (Coskun, 2015).

Individual usage of mortgage loans started in Turkey in the 60 s through a public bank. Emlak Bankasi provided 20-year mortgage loans with 60 and 66% interest rates, between 1962 and 1993 to individuals and housing cooperatives to purchase land and construct housing (Gurbuz, 2001). These interest rates were lower than the inflation rates between 1988 and 1993, during years when inflation rates were around 75% (Gurbuz, 2001). However, even though the mortgage interest rates provided by Emlak Bank were lower than the inflation until 1993, mortgage repayments led to hardships for many people (Gurbuz, 2001). State owned banks did not have enough financial resources to continue giving mortgage credits in the mid-90 s, while private banks were not willing to give mortgage loans (Gurbuz, 2001).

Alternatively, in 1984, TOKI, a state-owned AH institution was established (Gurbuz, 2001). Also in 2002, the assets out of banking operations and real estate of Emlak Kredi Bank were transferred to TOKI (TOKI, 2012). Since then, Emlak Konut GYO (Emlak Konut REIT) has developed and become one of the largest subsidiaries of TOKİ and began to produce housing for middle- and high- income groups (TOKI, 2012).

TOKI gave project loans with low interest rates to municipalities and cooperative housing companies; and TOKI produced AH only for low-income holders in 1984 (TOKI, 2012).

TOKI does not provide mortgage loans for home purchases, but TOKI maintains ownership of the home and provides opportunities for residents to make instalments for five to twenty-year periods, until the value of the home is paid in full and ownership is transferred to the resident (TOKI, 2016b). A draw is arranged if the number of applicants are greater than the housing units produced. The TOKI home ownership through instalment program offers a lower interest rate plan than mortgage loans for low, middle, and high-income groups. In 2011, TOKI’s interest rate for instalments for individuals was 7,96%, the rate was 5% in 2021. Private banks offered mortgage loans between 10 and 14% in 2011 and 18 to 20% in 2021 indicating high inflation rates (TCMB, 2021b; TOKI, 2021).

Mortgage policy deregulated in Turkey in 2007 that allowed financial leasing companies, commercial and public banks to provide mortgage loans for consumers (LawNumber5582, 2007). Therefore, the number of mortgage loan sources increased and led consumers to borrow with lower interest rates in comparison to the 90 s (Akçay, 2011; Gülter & Basti, 2014). However, to qualify for a mortgage loan, 50% or 60% of the total home price is required as a down payment, which is not affordable for low-income holders in Turkey (Akçay, 2011; Gülter & Basti, 2014).

## Methodology

A variation finding comparative case study method is used in this study. The study consists of country level policy and results comparison in Denmark and Turkey (Goodrick, 2014; Pickvance, 2001; Tilly, 1984). Financial crisis periods provided sufficient conditions to develop neoliberal housing policies supporting real estate specific economic growth (Goodrick, 2014; Pickvance, 2001; Tilly, 1984). Financial crisis periods occured in Turkey in 2001, 2007 and 2018, in Denmark in 2007. Macro-economic factors existing after the financial crisis, includeing unemployment rates, led the state to facilitate easy access to consumer loans to individuals with economic deficiencies.

A variation finding comparative case study was designed through arguments based on variables to explain neoliberal housing policies supporting financialization (Pickvance, 2001; Tilly, 1984). The comparison includes a pattern matching consisting of similar housing policy comparisons in two different countries (Goodrick, 2014). Also, background conditions and characteristics of cases were considered to organize data into categories (Pickvance, 2001; Tilly, 1984). Therefore, comparable units of data were divided into the categories: welfare systems, housing market types and tenure distribution, which are different in two countries (Goodrick, 2014; Tilly, 1984).

The comparison of neoliberal housing policies in the two countries suggests that despite varying country characteristics, states employ similar neoliberal housing policies to provide fast economic growth after financial crisis periods with financialization. Neoliberal housing policies included deductions of interest rates from income taxation, deregulation of rent policy and promotions for the growth of interest free mortgages in Denmark. In Turkey, state support for the housing boom was, provided through state subsidies given to people with conditions. Conditionalities included opening a bank account with the aim to enable homeownership and receive mortgage support with reductions of interest rates.

The main actors of developing and implementing housing policy and transforming to neoliberal housing policy include but are not limited to state, financial institutions, housing production companies, home owners and consumers. However, these actors, institutions, are sometimes blended in several ways and are not compared directly in this study. Instead, the decisions and impact of their choices are addressed in response to the desire of homeownership being challenged by responsibilities to mortgage debt and hidden factors of inequality, that are addressed throughout the paper.

### Data set, limitation of the data and the usage of data to define low-income groups

Gini coefficient levels, and the changing share of top 10% income holders, bottom 10% and top 1% in the national income were used to give information about the relationship between homeownership and wealth inequalities. Statistical sources were the World Bank, IMF, OECD, the Danish Statistics Institute (Statbank; Dst.dk), Turkish Statistics Institute (TUIK), statistics of the National Bank of Denmark (Nationalbanken) and the central bank of Turkey (TCMB). A research report of a private real estate consulting company, REIDIN, was used to gather Turkish housing prices increase statistics for the period before 2010 that do not exist in TUIK’s database. Statistical sources were listed below (Table 1),

**Table 1:** Data Sources

Sources	Income data	Housing data	Mortgage/loan
World Bank	10%, bottom 10% and top 1%;		
IMF			Household debts
OECD	Gini coefficient, share of top 10%, bottom 10% and top 1% income holders in the national income; Home ownership rates	Housing and rent prices	Inflation rates, debts
Statbank	Disposable income, Gini coefficient,	Home ownership rates	
TUIK	Disposable income, Gini coefficient,	Home ownership rates	Housing purchase numbers, household debts
National Central Banks			Housing purchase numbers, inflation rates
REIDIN		Turkish housing prices before 2010	

The assessment of poverty, the categorization of higher incomes and their relationships to homeownership are arranged as follows and data was provided from Statbank and TUIK. People earning below 60% of average national income are considered as low-income groups. This definition of low-income groups aligns with the poverty line for households below 60% of national average income as used by the Turkish Statistical Institute and the European Union Statistical Institute (Eurostat).

In Turkey, people earning between 60% and 120% of average income are defined as middle income; and people earning more than 120% of average income are defined as high income holders (TUIK, 2021a). However, in Denmark, the distribution of home ownership is based on seven different income amounts on the Danish national statistics database. The majority of the home ownership increase was among the top income groups, those with 180% of average income earning more than 600,000 DKK per year. Therefore, to compare with Turkish data, annual changes of average income levels for Denmark were calculated to find 60%, 120%, 180% of average income levels and more than 180% to adjust with the homeownership levels of these income holders.

There are limitations to accessing the data for both countries. Danish wealth inequality information is provided by the OECD and includes housing value calculations based on their value of purchase and taxation during a year (Balestra & Tonkin, 2018). There is no OECD database for Turkey regarding wealth inequality, which cannot be used for the Turkish case of this paper. However, Turkish national household disposable income data includes real estate total net income, which was used to assess wealth generation by homeownerships (TUIK, 2021a).

## Findings: The usage of mortgage in Denmark and Turkey

Amortization free interest-only loans in 2003 required borrowers to only pay the yearly interest rates (Bäckman & Khorunzhina, 2019). After 10 years, a mortgage holder is required to pay the remaining amount of the borrowed loan or initiate another interest only loan for a 30-year period (Bäckman & Khorunzhina, 2019). Therefore, a mortgage holder can delay the large share of debt for 30 years with only paying a monthly interest rate amount that causes household debt to increase. In Denmark, interest rates were 2.5% in 2015, and 65% of the population in Denmark used mortgages to purchase homes (Nationalbank, 2021; Worldbank, 2022b).

**Figure 3:**
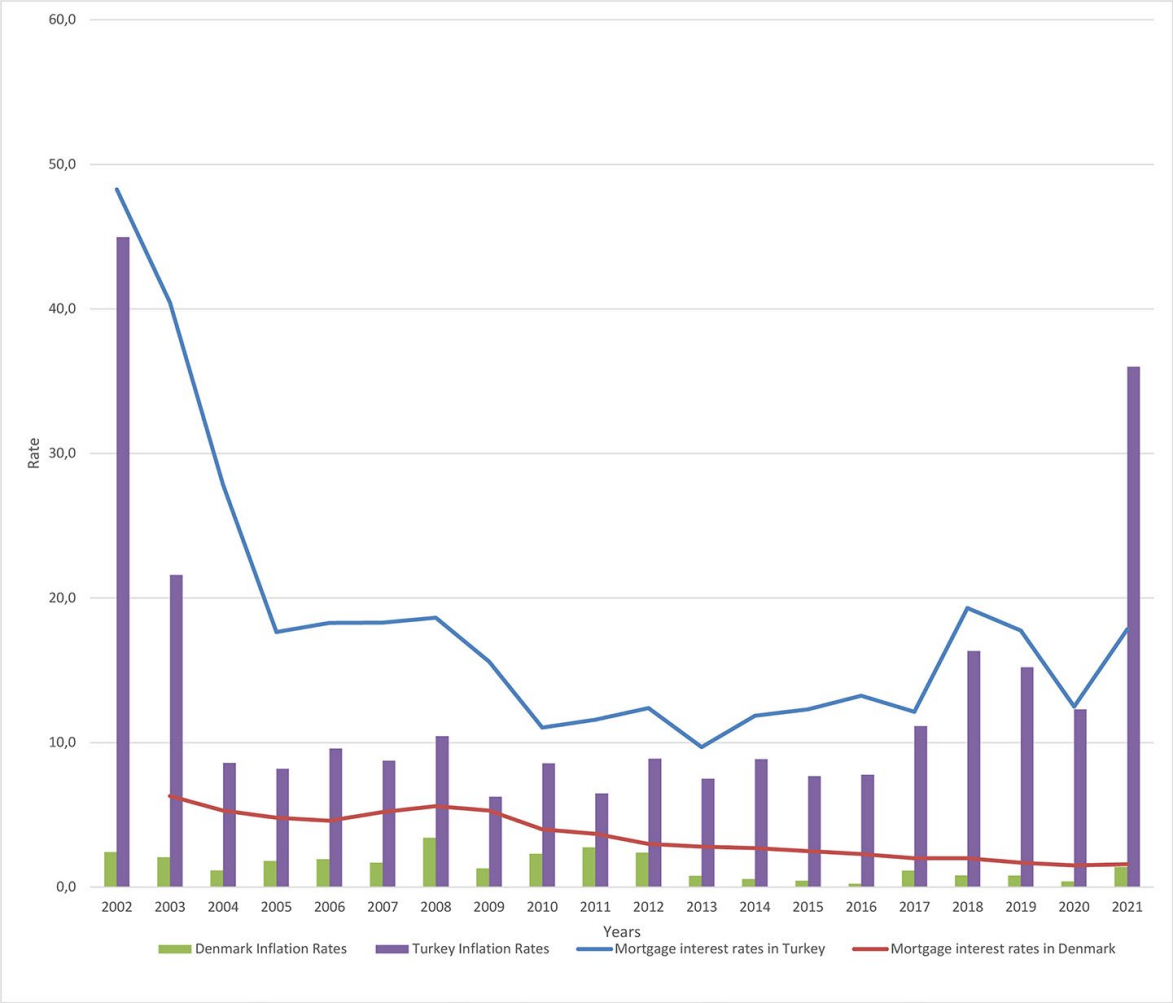
A Comparison of Mortgage Loan Interest Rates and Inflation Rates in Denmark and Turkey. Note: Interest rates of mortgage loans and inflation rates in Denmark and Turkey, 2002-2020 (Nationalbank, 2021; TCMB, 2022; Worldbank, 2022b).

Inflation rates in Turkey were almost 50% in 2002 causing banks to give mortgages with high mortgage interest rates, but then mortgage interest rates reduced to over 10%, as shown in Fig. 3 (Nationalbank, 2021; TCMB, 2022; Worldbank, 2022b). However, interest rates are still higher to pay back for many people. As a result, Turkey has the second lowest rate of residential mortgage loan usage among OECD countries that was 0.3% in 2005; later, in 2011, the rate of mortgage usage increased to 15% in GDP (Whitehead & Williams, 2017).

### Increasing number of home purchases in both countries

The real estate sector has become an investment instrument that benefits higher income earners who have greater capacity and access to finance (Forrest 2018), in Denmark. Hence, as a result of easy access to mortgage loans in Denmark, the number of home purchases peaked in 2005 but declined to their lowest level in 2011, then peaked again in 2017 (Statbank, 2019b). This decline in the number of purchased homes in 2011 was related to insolvent mortgage debts (Bäckman & Khorunzhina, 2019).

In Turkey, the welfare system has had the added responsibility to promote home ownership, through TOKI. TOKI, since 2004, has become the state-owned AH production coorporation that produces, rents and sells homes to Turkish citizens. Even though AH is not a part of the welfare system, it was placed at the heart of social policies in a letter of good will to the IMF requesting financial assistance (Bayirbağ, 2013). The IMF required Turkey to restructure its welfare system to receive aid (Bayirbağ, 2013). A fast-growing economy based on construction and housing boom stimulated Turkish citizens to become dependent on financialization by purchasing more than one home. Therefore, more than one million homes have been purchased not only from TOKI but also from private companies in Turkey annually since 2013 (TUIK, 2021b). High number of housing purchases is a characteristic of financialization in Turkey, which continued even during the period of the currency crisis of 2018 (TUIK, 2021b).

### Tax incentives embedded in homeownership promotions

Neoliberal housing policies included a freeze on housing taxation in 2001 that provided extra desire to become a homeowner in Denmark (Nationalbanken, 2019a, 2019b; Skat, 2016). Additionally, another property value tax reform (Forældrekøb) was redeveloped for parents purchasing a home if they rent it to their children, which has provided income tax reductions since 2016 (Nationalbanken, 2019a, 2019b; Skat, 2016). As a result, plunging house prices were observed in Denmark (ESRB, 2016; Nationalbanken, 2018).

An expansion of interest only loans led to overvalued homes in 2016, leading one-third of mortgage debtors to owe mortgage amounts that exceed the real value of their homes (ESRB, 2016; Nationalbanken, 2018). The same situation was previously observed during the financial crisis of 2007 in Denmark. This repitition indicates a pattern of risk of a possible financial crisis caused by overvaluation of housing (ESRB, 2016; Nationalbanken, 2018). However, Danish homeowners purchase and let homes to resell with the idea that homes will increase in value and become an opportunity to make wealth through homeownership with mortgage debts, which explains why Denmark has the second lowest rate of outright homeownership (Fuller et al., 2020; Petach, 2018; Ryan-Collins et al., 2017).

**Figure 4:**
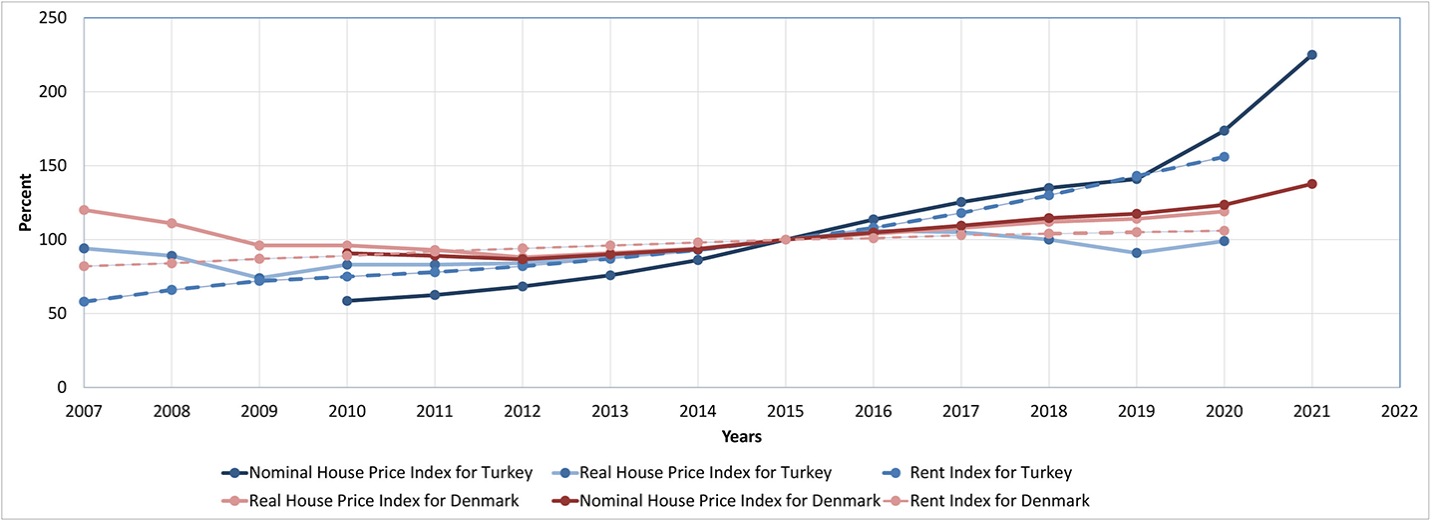
Real rent and house price indices (inflation rates are considered in housing price indices) for Denmark and Turkey (OECD, 2022; REIDIN, 2011)

Therefore, at the national level, real house prices in Denmark in 2019 almost reached those observed before the 2007 financial crisis, as shown in Fig. 4 indicating housing values and demand. Housing prices bottomed out in 2012, but increased again by 30% in 7 years. However, young and low-income households cannot even purchase homes due to higher housing prices in comparison to the past. In 2019, the number of single low-income holders in Denmark was more than halved in comparison to 2006 (Statbank, 2020).

Even though Denmark and Turkey have different housing market dynamics, price increases between 2012 and 2017 were similar particularly in their largest cities, Copenhagen and Istanbul. The Turkish construction sector receives the largest share of the GDP, 8.5% in 2016 (EUROSTAT, 2021b). In Turkey, due to increasing inflation rates, a downward trend in housing prices occurred between 2017 and 2019.

In Turkey, house prices increased between 2010 and 2016 by 28%, as shown in Fig. 4. Also, between 2010 and 2019 a housing boom occurred in Turkey, resulting from the remarkable growing number of housing construction permits given by municipalities (TUIK, 2021b). The Turkish state has played a part in the housing boom by promoting home ownership and increasing the housing construction activity of TOKI. Through state support, TOKI produces almost 9.2% of housing for low, middle and high income holders annually as a result of neoliberal housing policies (TOKI, 2016a; Turk, 2019).

Also, Turkish state developed VAT incentives temporarily; and has been providing grants for first-time home buyers who have opened a bank account since 2018. State reduced VAT from 18 to 8% during the sale of newly produced homes between 2018 and 2020 (LawNo:11674, 2018). Additionally, the state encouraged Turkish citizens to open bank accounts in public and private banks to purchase a house in 2018 when a currency crisis emerged (LawNo:7103-Annex:3, 2018). The state provided grants up to 25%, not exceeding 30,000 Turkish Lira in 2021, of the purchase price of a home; provided citizens kept the money in the bank for 3 years (LawNo:7103-Annex:3, 2018). In other words, the state grants provided the down payment for the home purchase, the new homeowner paid the mortgage payments while keeping their capital in the bank.

When the currency crisis was observed, the rate of mortgage use for home purchase decreased to 20% in 2018 (TUIK, 2021a). In the following years of the currency crisis, the state reduced interest rates temporarily on mortgages to 0.99% by Turkish central bank in 2019 and by public banks in 2020 (Aksam, 2019; Milliyet, 2020; Takvim, 2019). State owned banks were required to offer mortgages lower than private banks in 2020 (Akgündüz, Neef, Hacıhasanoğlu, & Yılmaz, 2021). The state grant support in down payments for home purchases coupled with the lowered mortgage loan interest rates increased the interest in becoming a homeowner in Turkey. Therefore, TOKI and related housing construction companies in municipalities were able to sell their products, homes produced before 2018. As a result, mortgage usage rates increased from 24 to 38% between 2019 and 2020 and led to housing price increases, as shown in Figure 4 (TUIK, 2021a).

The same strategy was used in 2009 when the central Turkish bank temporarily reduced interest rates to 0.99%, leading to a peak in mortgage usage between 2009 and 2010 from 4 to 40%, respectively (Takvim, 2009; TUIK, 2020, 2021a). State intervention through the Turkish central bank to reduce the interest rates of mortgage loans is a direct state support of financialization through homeownership. Furthermore, the target of reducing interest rates was also to support construction companies to sell more homes to save them from a possible bankruptcy due to reduced rates of housing purchase in crisis periods, and for national economic growth (Gürakar & Bircan, 2016).

### Homeownership promotion led to changes in household indebtedness and wealth

**Fig. 5:**
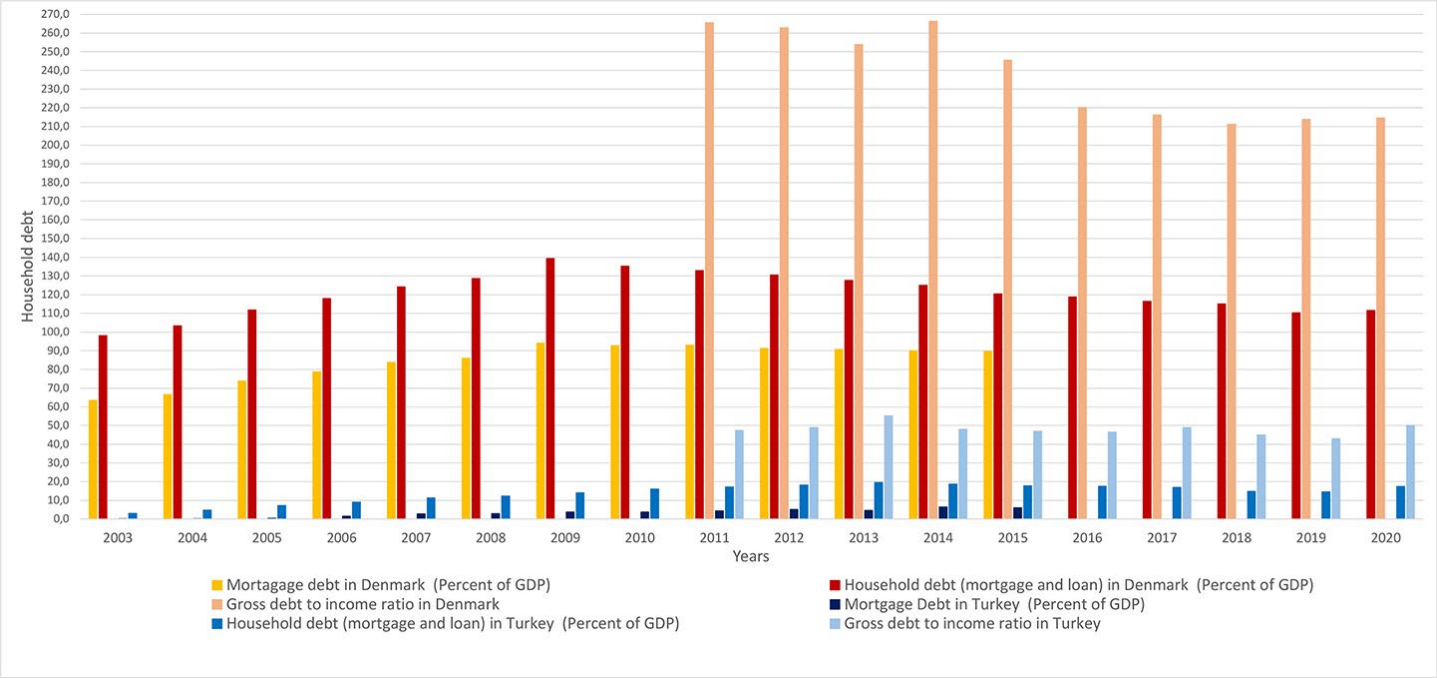
Household debt and mortgage debt to the GDP ratio trend (%) (DanmarksNationalbank, 2021b; IMF, 2018; TBB, 2019; TCMB, 2021a; Whitehead & Williams, 2017)

In Denmark, the allowance of interest only mortgages loans, particularly in large cities of Denmark such as Copenhagen, motivated people to borrow more than their income and make mortgage payments toward interest only for certain time periods (H. T. Andersen & Winther, 2010; Nationalbanken, 2018, 2021). People are allowed to borrow two or three times more than the household disposable income and debt to GDP levels increased as shown in Fig. 5 (Nationalbanken, 2018). Denmark has become the highest indebted OECD country since the mid-90 s (OECD, 2020b).

55.5% of homes were purchased by mortgages in 2001, and this increased to 89.7% in 2015 (Whitehead & Williams, 2017). The proportion of household debt to net GDP increased from 64% in 1994 to 140% in 2007, and to 240% in 2017; the mortgage debt is the largest expense of household debt in Denmark, as shown in Fig. 7 (Nationalbanken, 2018). In 2017, highest income holders in Denmark; are placed in the top three 10% income deciles and had almost half of the total mortgage household debts of the country, leading high-income holders have the highest median debt to income ratio, which was 256% (Nationalbanken, 2018). The remaining income deciles had half of household mortgage debt between 2002 and 2017, showing the distortion of the balance in wealth accumulation and growing household debts at the national level (Nationalbanken, 2018).

In contrast to Denmark, Turkey is the second lowest mortgage indebted country in the share of GDP in the OECD after Russia, due to high inflation rates in Turkey that requires people to pay higher monthly mortgage payments, as of in 2016 (Whitehead & Williams, 2017). Therefore, many people are not financially eligible for mortgage loans (Akçay, 2011). As a result, home purchases with mortgage loans have remained low in Turkey; instead home purchases are made through reliance on family equity financing (Akçay, 2011).

However, mortgage law deregulations in 2007, allowed middle income people to become eligible to purchase homes with mortgages (Akçay, 2011). Debt to disposable income ratios of Turkish citizens have increased over years, from 3% in 2002 to 23% in 2007, jumping to 36% in 2009, growing to 52% in 2013 (Soral, 2015). Debt to disposable ratios show the ability of people to pay back their loans and in Turkey households spent half of their income to cover their debts in 2020, as shown in Figure 5, above (TCMB, 2021).

### Changing rates of homeownership among different income groups

**Fig. 6:**
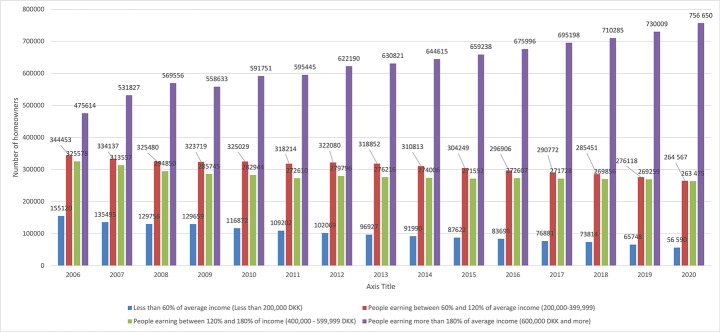
Changes in the number of homeownerships among different income holders in Denmark (Statbank, 2022)

In Denmark an increase in home ownership only occurred among very high-income holders, and peaked from 2006 to 2008 at 6,6%. In Turkey, the peak was between 2007 and 2008 at 2.4%, during the financial crisis. These increases reflect that, financial crisis periods do not affect higher income^4^ holders to mitigate their home them purchases. In Denmark, the highest income group earning more than 600,000 DKK annually, as shown in Fig. 6, experienced a remarkable increase in home ownership between 2007 and 2019 (Statbank, 2020). On the other hand, all other income groups experienced declining home ownership in the same period (Statbank, 2020). However, the most dramatic decline in home ownership was among the lowest income holders (Statbank, 2020).

**Fig. 7:**
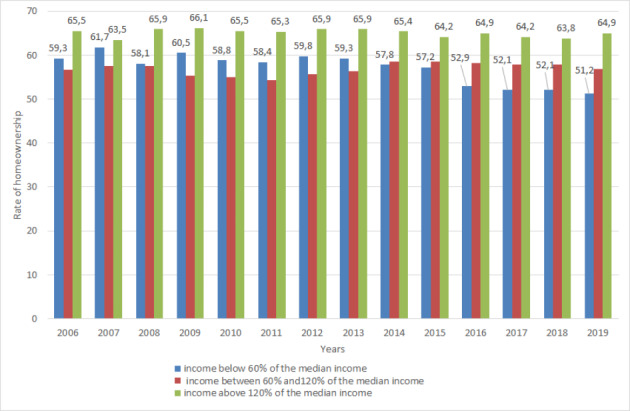
Homeownership rate changes among low, middle- and high-income groups in Turkey (TUIK, 2021a)

Similar to Denmark, in Turkey low income groups had the highest level of homeownership losses, as shown in Fig. 7 (TUIK, 2021a). In Turkey, high-income groups had an increase in home ownership during the same years, 2007 through 2019, even though they had household debts (Egilmez, 2018; TUIK, 2021a). Mortgage promotions being mostly beneficial to middle-income holders explains the growing number of home ownership among that income group (Egilmez, 2018; TUIK, 2021a). In 2019 after the currency crisis, the homeownership rate of middle-income holders had a dramatic decline while high income holders increased their homeownership rates. Different than Denmark, in 2020, while middle and high income owners had a decline in homeownership rates, low-income holders increased their homeownership rates.

### Wealth inequalities

**Fig. 8:**
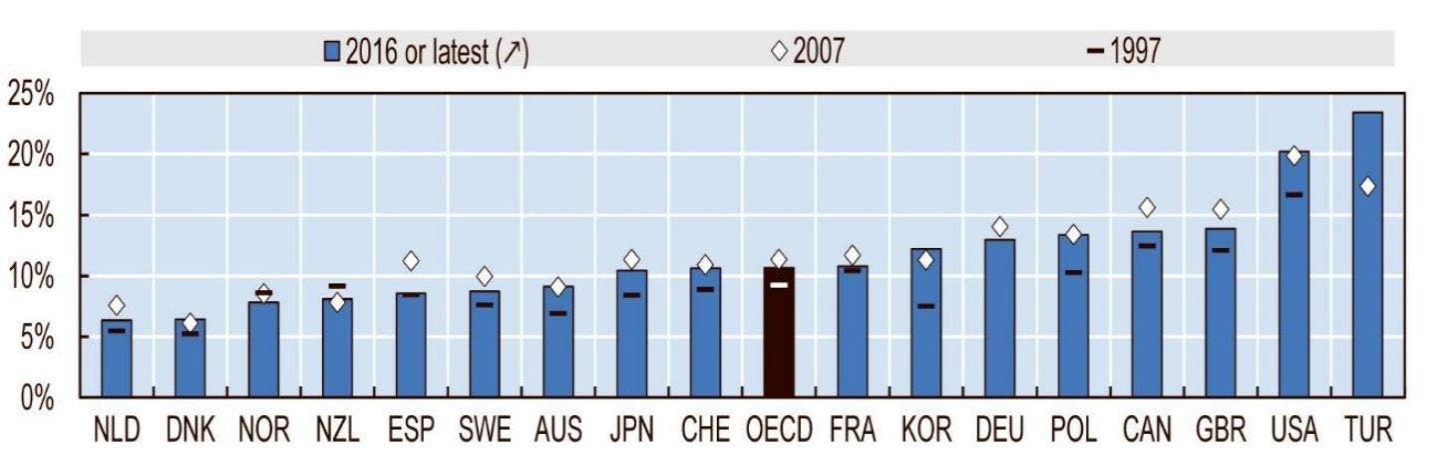
Changing income share of top 1% in OECD countries (p. 58) (OECD, 2018b)

In both countries, the wealth growth of top 1% holders between 2007 and 2016 is larger than GDP growth meaning that the majority of the income returned with economic activity is gathered by top 1% income holders. In Denmark, the income share of the top 1% was one of the lowest shares and increased by 2% between 2007 and 2016, as shown in Fig. 8 (OECD, 2018b). In Turkey, the share of income of the top 1% income holders was the highest among OECD countries (p. 58) after the financial crisis of 2007 and the increase was from 17 to 24% in 2016 (OECD, 2018b).

**Fig. 9:**
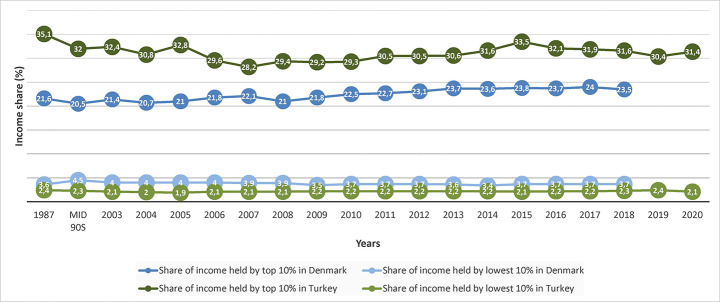
Income share held by top and bottom 10% income holders in Denmark and Turkey between 1987 and 2018 (TUIK, 2021; Worldbank, 2021)

Denmark had the third highest wealth inequality after the US and the Netherlands among OECD countries in 2015 (Balestra & Tonkin, 2018). In Denmark, the net wealth of the top 10% income holders was 64% in 2015, while their income share was 23.8%, which is explained as explained in an OECD report as being due to the earned wealth from housing through increasing housing prices (p. 25) (Balestra & Tonkin, 2018). The share of top 10% holder’s income in the GDP grew one year after the financial crisis of 2007, which was more than the increase observed between 1987 and 2008 in Denmark. On the other hand, the share of bottom 1% income in Denmark has been declining dramatically since the mid-90s (Worldbank, 2022a; Fig. 9).

In Turkey, the share of top 10% income holders increased constantly after the financial crisis of 2007, more than the period between mid-90 s and 2007 (TUIK, 2021; Worldbank, 2021). Between 2015 and 2018, the share of income of top 10% holders declined. On the other hand, the income share of bottom 10% holders increased slightly, reaching the same share in the mid-90 s (TUIK, 2021; Worldbank, 2021). Particularly, between 2016 and 2017 housing prices and rent amounts peaked (TCMB, 2018a). Turkey had the highest share of income of top 1% holders in the share of GDP by 24% of OECD countries in 2016 (OECD, 2018b). Only Turkey, out of all OECD countries, increased wealth among 1% income holders after 2007 (OECD, 2018b), showing that housing production not only lifted economic growth but also wealth growth.

## Concluding discussion

This paper answered how and why neoliberal housing policies have changed homeownership rates among different income holders in the surge of household debts in Denmark and Turkey. A reason neoliberal housing policies were implemented was to facilitate fast real estate-based economic growth after economic crisis periods in both countries, and led to an increase in supply and demand of housing. The housing market became a regulator to provide fast economic growth for governments and investors to generate profit. Mortgage loans became more accessible and deregulations in housing markets facilitated people borrowing mortgages to purchase homes and housing companies to produce. As a result, financialization and consumption have become a deterministic means to regulate the economy.

Deregulations and financialization had an effect on housing prices that have been increasing in both countries. Neoliberal housing policies provided wealth generation through homeownership to individuals in high and middle-income groups and to housing production companies. Facts of wealth inequality show that while higher income groups accumulate wealth particularly with homeownership, lower income groups become dispossessed through declining homeownership rates. In both countries, home ownership rates among low-income groups declined between 2007 and 2018.

In Denmark, many people who do not have very high incomes do not own their home outright. Some Danish homeowners often do not own their homes outright because they sell their homes to profit from increasing housing prices to purchase another home with another mortgage loan or to have cash value of housing through home equity withdrawal. The overall result of increasing house prices led the country to have a high rate of wealth inequality and one of the lowest rates of outright homeownership in 2015.

In Turkey, between 2007 and 2018, middle income holders had the largest increase in homeownership showing that mortgage loans promotions have primarily benefited middle income holders. The number of low-income homeownerships declined abruptly in 2019 while homeownership of high-income holders increased. However, there is no data for outright ownership in Turkey. The growing number of homes sold each year and governmental promotions to attract people to use mortgage loans, stimulate people to purchase own more than one home, with at least one used as an income earning asset investment.

In Copenhagen and Istanbul housing prices increase faster than the other cities that affect young people starting their careers and looking for housing. Therefore, the housing affordability problem has been growing for young couples.

Since 2018 and into the recent decade, new financial crises have emerged alongside the global economy affected by COVID 19 and rising inflation. The patterns of housing policy adjustments aimed to counter national economies continue to follow asset-based welfare models in the housing market. In Turkey, to contrast increasing national inflation rates and economic situation of Turkish citizens, in 2018 the government offered Turkish citizenship to foreigners who purchase a home in Turkey for 250,000 USD leading to a 62% increase in nominal housing prices in Turkey between 2019 and 2022. In both countries the number of purchased homes increased in the last 2 years in parallel to increasing housing prices. Financialization supported by governments through deregulations is used to create fast economic growth and motivate people to purchase homes. The neoliberal deregulations embedded with new forms of financialization, often supported by governments, have long term effects. One of these effects are observed by the increasing household debts. Another effect is observed in large cities with increasing inequalities among not only the income of people but also their location in the cities. Large cities with growing wealth inequalities based on homeownership have created a divide between homeowners and tenants, as observed with housing segregation.

As long as neoliberal policy promotes wealth to be created through and dependent on homeownership, otherwise referred to as asset based welfare, there will be no homes for poor men. People who are homeowners seek for profit with increasing prices of housing and rents while people who are not homeowners seek to save more money to afford down payment of mortgage or rents. The rate of young, single or low-income households being a homeowner was more than halved in 2019 in comparison to 2006. As long as housing prices continue to increase without equivalent wage increases there will be less opportunity for many young people with middle and low income to become homeowners in the future.

## Footnotes

1 Gini coefficient in income inequalities gives an overall information about income deviation from the mean, because the highest and lowest income were removed from calculation and is sensitive to measure changes in middle incomes. However, a complete picture of income growth is also related to increasing economic gains of top 1% and 10% income holders that is based on cumulation of financial and nonfinancial assets such as net value of housing or inheritance. (Fuller et al., 2020; Ryan-Collins et al., 2017)

2 Wealth consists of financial and non-financial assets that have economic value and are legalized by ownership rights (Balestra & Tonkin, 2018) The value of assets calculated after subtracting outstanding liabilities include non-financial assets such as dwellings and other real estate, valuables, vehicles and non-financial assets such as deposits in banks, shares in companies and revenues gathered with engagements in pension funds (Balestra & Tonkin, 2018).

3 “Social expenditure is the provision by public (and private) institutions of benefits and financial contributions targeted at households and individuals in order to provide support. Such benefits can be cash transfers, or can be the direct (“in-kind”) provision of goods and services” (OECD, 2001).

4 In Turkey, people earning more than 120% of average income are defined as high income holders in this article. In Denmark, people earning between 120% and 180% of average income are defined as high income holders and people who have 180% of average income are defined as very high-income holders.
